# Cloning and functional verification of a porcine adipose tissue-specific promoter

**DOI:** 10.1186/s12864-022-08627-0

**Published:** 2022-05-24

**Authors:** Dawei Zhang, Liangcai Shen, Wenjing Wu, Keke Liu, Jin Zhang

**Affiliations:** 1 College of Biological, Chemical Sciences and Engineering, Jiaxing University, Jiaxing, 314001 China; 2 College of Agronomy and Biotechnology, Hebei Normal University of Science and Technology, Qinhuangdao, 066000 China

**Keywords:** Adipose tissue-specific promoter, *LGALS12*, Pig, *Sus scrofa*

## Abstract

**Background:**

Fat deposition is an important economic trait in pigs. In the past decades, many genes regulating porcine fat deposition were identified by Omics technology and verified by cell biology studies. Using genetically modified pigs to investigate the function of these genes in vivo is necessary before applying in breeding. However, lack of tissue-specific promoters of pigs hinders the generation of adipose tissue-specific genetically modified pigs.

**Results:**

In order to identify a porcine adipose tissue-specific promoter, we used the software Digital Differential Display (DDD) to screen 99 genes highly expressed in porcine adipose tissue. GO and KEGG enrichment analysis indicated that the 99 genes were mainly related to lipid metabolism. Q-PCR proved that *LGALS12* was an adipose tissue-specific gene. Five truncated fragments of the *LGALS12* promoter were cloned and the 4 kb fragment (L-4 kb) exhibited a high level of promoter activity in adipocytes and no promoter activity in non-adipocytes. Following co-transfection with adipogenic transcription factors, the promoter activity of L-4 kb was enhanced by PPARγ, C/EBPβ, and KLF15, whereas it was suppressed by KLF4. Finally, we demonstrated that L-4 kb can drive *APOR* gene expression to exert its function in adipocytes.

**Conclusions:**

This study demonstrates that porcine *LGALS12* is an adipose tissue-specific gene, and identified the 4 kb fragment of *LGALS12* promoter that exhibited adipocyte-specific promoter activity. These results provide new evidence for understanding porcine fat deposition and a promoter element for adipose tissue-specific genetic modification in pigs.

**Highlights:**

Identified porcine *LGALS12* as an adipose tissue-specific gene.

Truncated *LGALS12* promoter (L-4 kb) showed adipose tissue-specific promoter activity.

Identified transcription factors involved in the regulation of L-4 kb promoter activity.

**Supplementary Information:**

The online version contains supplementary material available at 10.1186/s12864-022-08627-0.

## Background

Genetically modified (GM) pigs are becoming increasingly important for both breeding and biomedical research [1]. For breeding purposes, GM pig research has focused on economically important traits, such as the growth rate [2], meat quality [3], disease/stress resistance [4] and feed conversion efficiency [5]. Although genetic modifications have not yet made real contributions to pig breeding, these methods have surpassed traditional breeding methods with rapid development and impressive results in a short period. For biomedical purposes, disease models have been established in pigs by genetic manipulation of key genes in disease pathways, which might offer a better representation of human pathology than rodent models for certain disease contexts [6]. Furthermore, pigs are also considered to be the best source of organs for xenotransplantation, due to their anatomical and physiological analogy to humans [7]. In the past decade, gene editing technology represented by CRISRP/Cas9 has developed rapidly, and has greatly facilitated the generation of GM pigs. However, whole-body gene-editing might cause unexpected side effects, which makes it different to obtain healthy offspring for study. In these cases, tissue-specific GM animal models are necessary. Hundreds of tissue-specific GM rodent models have been reported, but only a few have been reported in pigs. The main reason is the lack of tissue-specific promoters for pigs.

Digital differential display (DDD) is a free software for comparing expression profiles among the different library pools in UniGene (finished by 2012-4-26) [8], offering a quick method to identify genes with expression levels that differ between different tissues, stages, or conditions in organisms [9, 10]. In this study, pig adipose tissue-specific genes were screened by DDD analysis and verified by Q-PCR analysis. *LGALS12* showed the best specificity and its promoter was cloned. Furthermore, the transcriptional factors involved in *LGALS12* regulation were identified, which makes it possible to generate adipose tissue-specific GM pigs.

## Results

### Screening of genes preferentially expressed in porcine adipose tissue

DDD analysis was performed by comparing porcine adipose tissue libraries with porcine non-adipose tissue libraries including intestine, kidney, longissimus, lung, muscle, ovary, spleen, testis, thymus and uterus. A total of 5 adipose tissue libraries with 33,401 ESTs (pool A) versus 17 libraries of non-adipose tissues with 132,632 ESTs (pool B) were included in this analysis. The software identified 99 genes/transcripts with 10-fold higher expression levels in adipose tissue than in non-adipose tissues (Table S2).

**Fig. 1:**
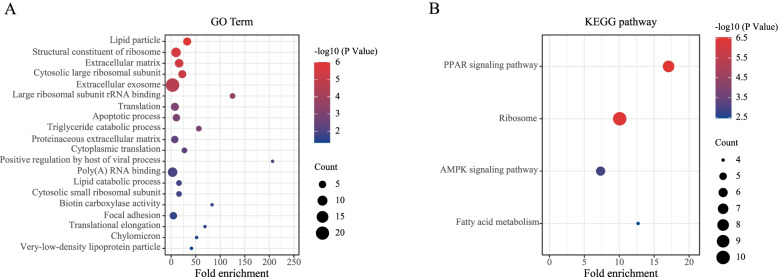
Functional enrichment classification of genes preferentially expressed in porcine adipose tissue. (**A**) Significant GO terms and (**B**) significant KEGG signaling pathways

These 99 genes/transcripts were subjected to Gene Ontology (GO) analysis to predict their potential functions. The top 20 enriched categories related to biological processes with *P* < 0.05 were listed (Fig. 1A). The results were mainly related to lipid metabolism (Lipid particle, Triglyceride catabolic process, Lipid catabolic process and Very-low-density lipoprotein particle) and ribosome function (Structure constituent of ribosome, Cytosolic large ribosomal unit, Large ribosomal subunit rRNA binding, Translation, Cytoplasmic translation and Cytosolic small ribosomal subunit). KEGG enrichment analysis was also conducted to identify the involved pathways (Fig. 1B). Four pathways, PPAR signaling pathway, Ribosome, AMPK signaling pathway and Fatty acid metabolism, were enriched, all of which are highly associated with lipid metabolism.

### *LGALS12* exhibited the highest specificity for porcine adipose tissue

**Fig. 2:**
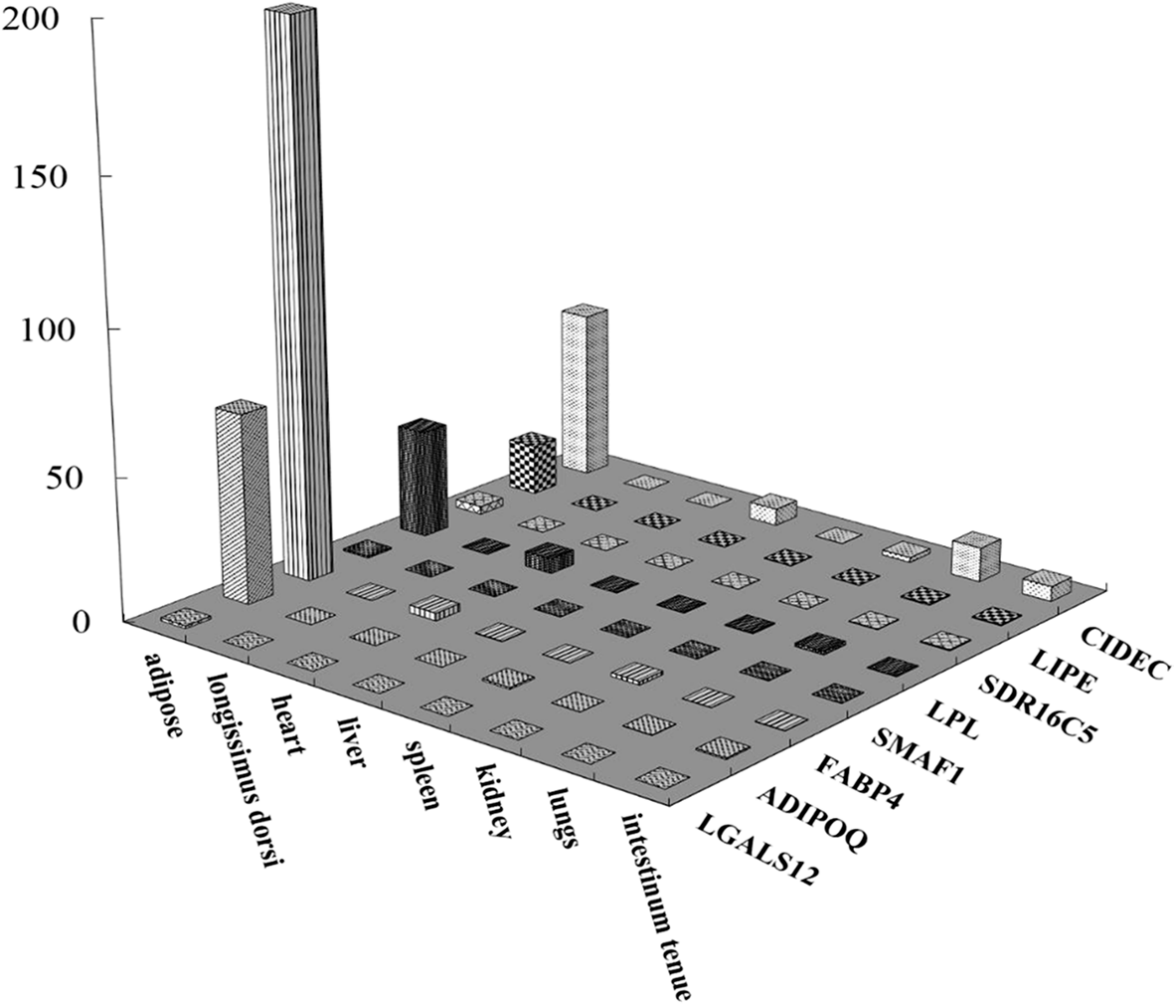
Expression patterns of eight genes with more than 30-fold change across different pig tissues. The expression level of each gene was assessed by Q-PCR, the fold-changes were calculated using the 2^−*ΔΔCt*^ method

In order to confirm the results of bioinformatic analysis, 8 genes with more than 30-fold change were verified by Q-PCR analysis (Table 1 and Fig. 2). All 8 genes were preferentially expressed in adipose tissue, but their expression profiles varied significantly. The relative expression level of each gene in adipose tissue was compared, and was found to be in the order *FABP4*, *ADIPOQ*, *CIDEC*, *LPL*, *LIPE*, *SDR16C5*, *LGALS12* and *SMAF1*. The expression of the most highly expressed gene *FABP4* was 325 times higher than that of the least highly expressed *SMAF1*. The tissue expression patterns of these genes were analyzed and five genes, *CIDEC*, *LPL*, *LIPE*, *SDR16C5* and *SMAF1*, were detected in more than three tissues, although the expression levels in non-adipose tissue were very low. *FABP4* and *ADIPOQ* were detected in one tissue other than adipose tissue with very weak signals. *LGALS12* was specifically expressed in adipose tissue and was not detected in any other tissues. Therefore, *LGALS12* exhibited the best specificity for porcine adipose tissue. However, few reports on porcine *LGALS12* were found in the literature.

**Table 1:** Eight Genes Preferentially Expressed in Porcine Adipose Tissue

UniGene ID	UniGene name	gene symbol	fold changed in DDD analysis (pool A/pool B)
Ssc.1089	Fatty acid binding protein 4, adipocyte (FABP4)	*FABP4*	250
Ssc.18549	Adiponectin, C1Q and collagen domain containing (ADIPOQ)	*ADIPOQ*	170
Ssc.6784	Lipase, hormone-sensitive (LIPE)	*LIPE*	160
Ssc.16335	Lipoprotein lipase (LPL)	*LPL*	110
Ssc.49679	Lectin, galactoside-binding, soluble, 12 (LGALS12)	*LGALS12*	70
Ssc.21815	Cell death-inducing DFFA-like effector c (CIDEC)	*CIDEC*	35
Ssc.48111	Short chain dehydrogenase/reductase family 16C, member 5 (SDR16C5)	*SDR16C5*	30
Ssc.87912	Small adipocyte factor 1 (SMAF1)	*SMAF1*	30

### Cloning and activity analysis of the truncated *LGALS12* promoter

**Fig. 3:**
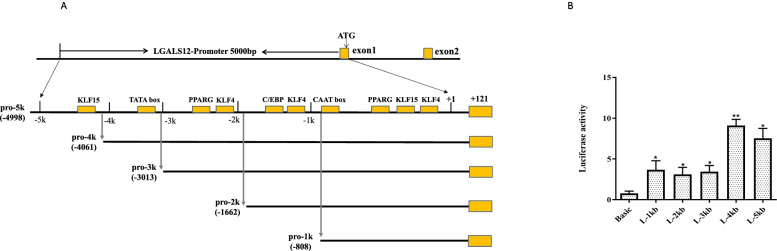
Promoter cloning and activity analysis of the *LGALS12* gene. **A** Schematic overview of the *LGALS12* promoter region. **B** Reporter activity of *LGALS12* promoter segments with different lengths in preadipocyte cell line 3 T3-L1. *p < 0.05, **p < 0.01

To obtain an adipose tissue-specific promoter, a 5 kb genome sequence upstream of the *LGALS12* start codon was analyzed. Five core promoter regions with scores over 0.85 were predicted (Table 2). Binding sites for adipogenic transcriptional factors were also predicted (Fig. 3A). Based on this analysis, the five truncated fragments indicated in Fig. 3 were cloned and named L-1kp, L-2 kb, L-3 kb, L-4 kb and L-5 kb. The fragments were used to construct the dual luciferase reporter vectors pGL3-L-1 kb/2 kb/3 kb/4 kb/5kB, respectively. Vectors with each truncated promoter were introduced into preadipocyte cell line 3 T3-L1 (day 4 of differentiation induction) and non-adipocyte line 293 T. The dual luciferase reporter assay was conducted to compare the promoter activity of each fragment (Fig. 3B). L-1 kb exhibited promoter activity in both cell lines, but the other four fragments showed promoter activity only in differentiated 3 T3-L1-derived adipocytes. L-4 kb showed the highest activity.

**Table 2:** The Prediction of the Core Promoter of the *LGALS12* Gene

Position(bp)	Score	Core promoter sequence
515–565	0.95	GCACTTGGGATCGAATTTCCCCATACAAAACGCCTTCAGC**C**ACCCAGCTC(− 1388 to − 1425)
672–722	0.87	CAGAATCACCAGGAGACTGTGTTAAAATGCTGATTCCTGG**G**CCCTGCCAC(− 1231 to − 1268)
82–1032	0.87	AAGGTGAGAGGTGGTGCTGCATTTTAAAGGGGAAGATTTG**T**TGGAATCTG(− 922 to − 958)
1157–1207	0.98	CGCCACTGTGGTCACCCTATCTATATACGGATGACCCAAG**G**TAAGCAGCT(− 746 to − 783)
1385–1435	0.90	TGAAATGGAGCCCCAGAGGATTATAAAACTTGCCAAAGAG**A**GAAGAGTGG (− 519 to − 555)

### Identification of transcriptional factors affecting *LGALS12* promoter activity

**Fig. 4:**
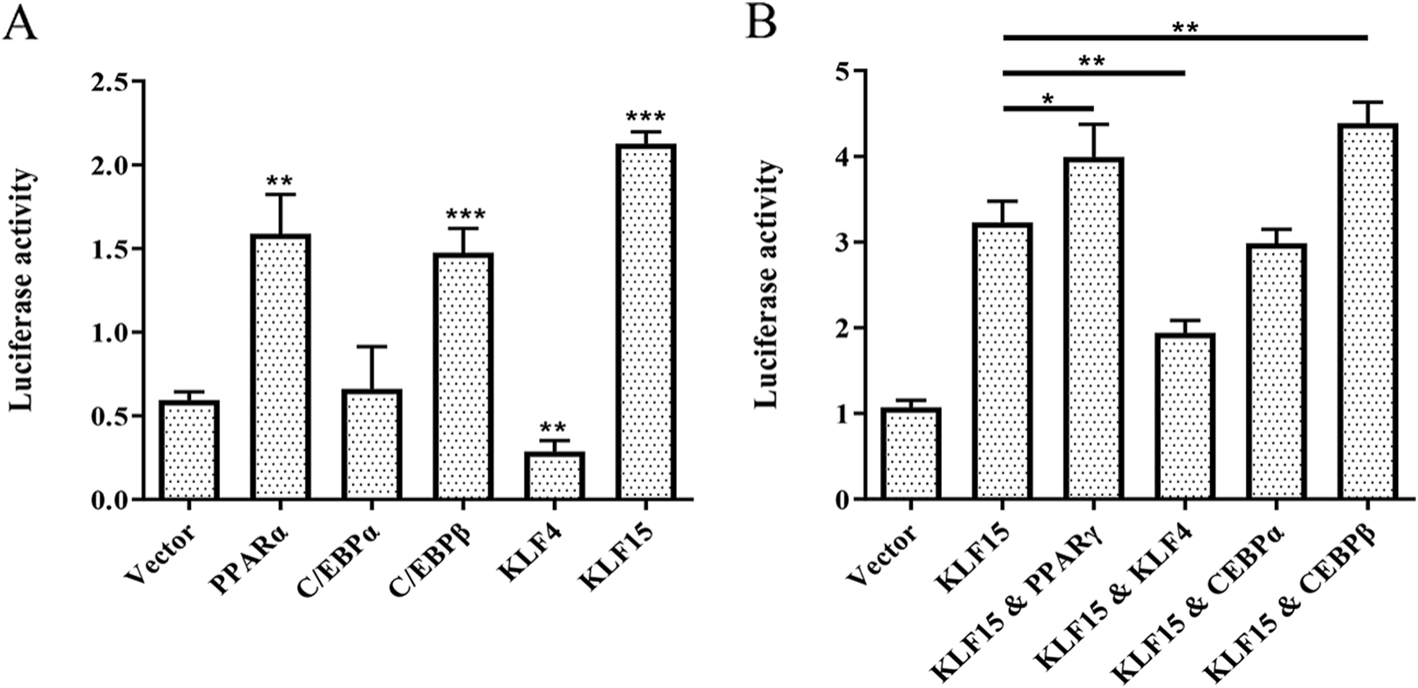
Screening of transcription factors that affect the activity of the *LGALS12* promoter using a dual-luciferase reporter assay. **A** L-4 kb co-transfected with each transcription factor individually (PPARγ, KLF4, CEBPα & CEBPβ). **B** L-4 kb co-transfected with the indicated combination of transcription factors. *p < 0.05, **p < 0.01, ***p < 0.001

In order to screen the transcription factors affecting the activity of the *LGALS12* promoter, 293 T cells were co-transfected with pGL3-L-4 kb and plasmids encoding five transcription factors, *C/EBPα*, *C/EBPβ*, *PPARγ*, *KLF4* and *KLF15*. The effects of each transcription factor on the activity of the pGL3-L-4 kb promoter were analyzed using a dual luciferase reporter assay (Fig. 4A). The promoter activity of pGL3-L-4 kb was enhanced by PPARγ, C/EBPβ, and KLF15 while being suppressed by KLF4. In order to assess the combined effects of transcription factors, *KLF15* was chosen as the highest promoting effector for co-transfection with *C/EBPα*, *C/EBPβ* and *PPARγ*, respectively (Fig. 4B). It was found that the transcriptional activity of pGL3-L-4 kb was improved by two combinations, KLF15 and C/EBPβ, as well as KLF15 and PPARγ. C/EBPα showed no effect on pGL3-L-4 kb in all assays.

### Functional verification of *LGALS12* promoter activity

**Fig. 5:**
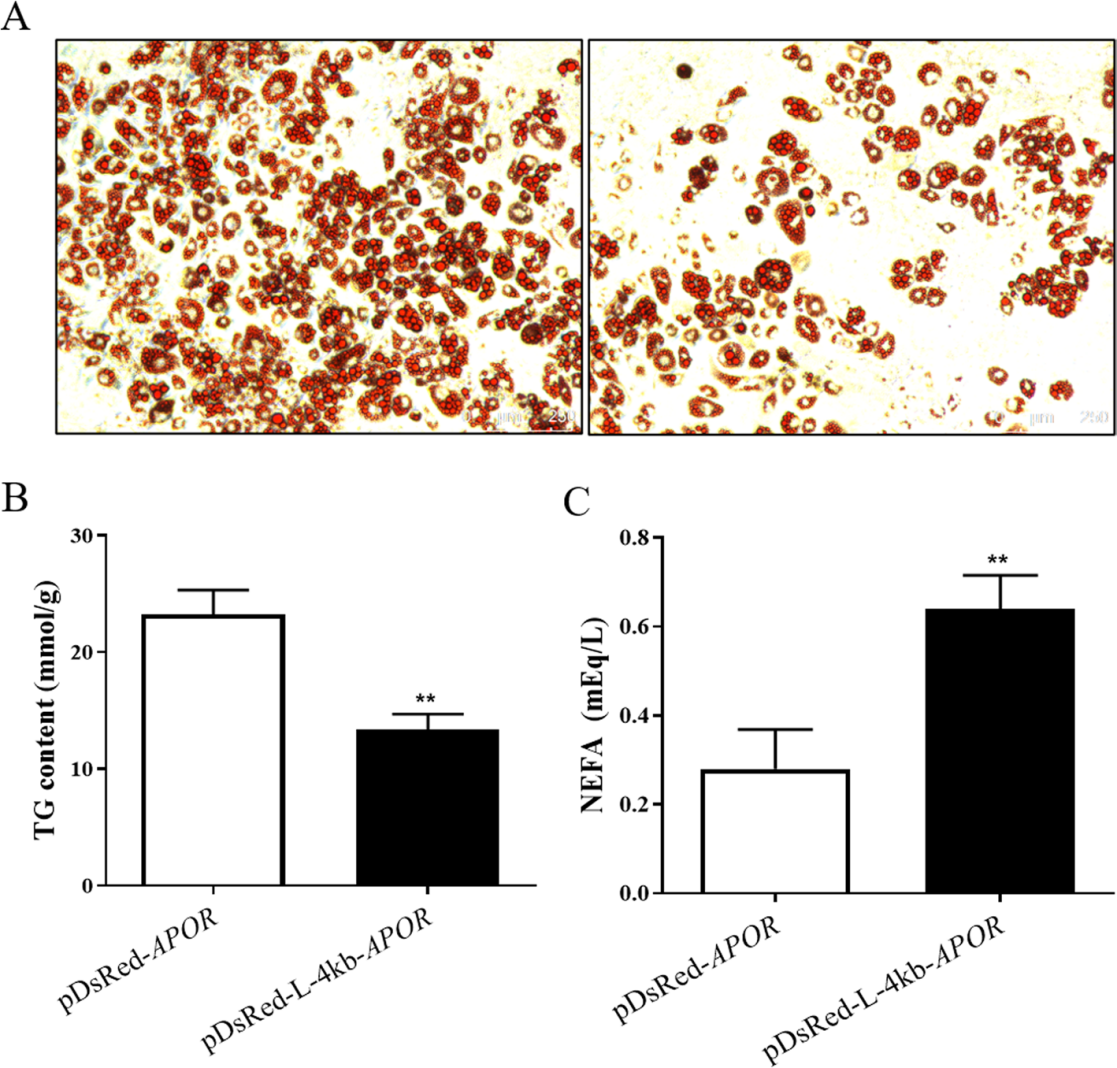
Functional verification of *LGALS12* promoter activity in 3 T3-L1 cells. (**A**) Oil red O (OR) staining indicated that *APOR* expression driven by L-4 kb influenced lipid accumulation. (**B**) Intracellular triglyceride analysis and (**C**) free fatty acid levels in the culture supernatant were consistent. **p < 0.01

To verify the function of *LGALS12* promoter activity, the L-4 kb fragment was cloned into the multiple cloning sites of the vector pDsRed-Express-N1, resulting in pDsRed-L-4 kb. The pDsRed-Express-N1 vector contains a red fluorescent protein (RFP) reporter gene without a promoter, which is used to detect promoter activity of cloned segments. Apolipoprotein R (APOR) can promote lipolysis in adipocytes and its coding sequence without a stop codon was cloned in to the vector downstream of the L-4 kb sequence, resulting in pDsRed-L-4 kb-*APOR*. The red signal could be seen when 3 T3-L1 cells were transfected with pDsRed-L-4 kb-*APOR*, while no signal was detected in the control group transfected with pDsRed-*APOR*. After 8 days of differentiation, oil red O staining and triglyceride analysis were conducted. Cells transfected with pDsRed-L-4 kb-*APOR* showed lower levels of lipid accumulation than control group (Fig. 5A and B). Furthermore, the group transfected with pDsRed-L-4 kb-*APOR* exhibited higher levels of free fatty acids in the culture supernatant (Fig. 5C). All these data proved that L-4 kb possesses promoter activity and can drive a gene to play its function in adipocytes.

## Discussion

Adipose tissue performs various physiological functions, including storing excess energy as fat, protecting inner organs from physical impact, keeping warm and secreting adipokines. Due to their highly developed adipose tissue, pigs are regarded as an ideal model for studying adipogenesis. Many important regulatory genes (including non-coding RNAs) for porcine fat accumulation have been identified by Omics technology and verified by cell biology studies in the past decades. Genetically modified (GM) pigs will be a powerful tool to unveil the functions of these genes and investigate possible side effects in vivo, which is necessary before application. However, the lack of porcine adipose tissue-specific genetic elements hinders the adipogenesis research based on tissue-specific GM pigs. There have been hundreds of research papers on adipose tissue-specific deficient/transgenic mice, but none on pigs so far. In this study, 99 genes/transcripts were identified highly expressed genes in porcine adipose tissue. GO analysis and KEEG enrichment indicated that the 99 genes were mainly related to lipid metabolism. By Q-PCR analysis, *LGALS12* were proved to be adipose tissue specific. According to bioinformatic analysis, five truncated fragments of the *LGALS12* promoter were cloned and the 4 kb fragment (L-4 kb) exhibited adipose tissue-specific promoter activity. Mechanistically, the promoter activity of L-4 kb was enhanced by PPARγ, C/EBPβ, and KLF15, while it was suppressed by KLF4. Finally, we proved that L-4 kb can drive *APOR* gene expression to play its function in adipocytes.

With the development of Omics technology, a large amount of data has been accumulated in the field of biology, and making efficient use of these data is a focus of research. Digital Differential Display (DDD) is a free online tool that can be used to compare EST-based expression profiles among different libraries, or pools of libraries, represented in UniGene [8], which is easy to learn and use. It can quickly screen the target gene set in the database, and is mainly used to screen genes with time- or space-specific expression [11, 12]. We used DDD to screen 99 genes out of 33,401 ESTs and focused on the top eight genes. All these eight genes were preferentially expressed in porcine adipose tissue, which indicated that DDD analysis could efficiently provide useful information.

According to Q-PCR analysis, we identified *LGALS12* as a porcine adipose tissue-specific gene. *LGALS12* belongs to the galectin family, which possesses conserved carbohydrate-recognition domains (CRDs) [13]. *LGALS12* was firstly reported in 2001 by two research groups independently [14, 15]. In 2011, one of the two groups proved that *LGALS12* deficiency promoted lipolysis in knockout mice [16]. We found that *LGALS12* was a key molecule in porcine fat deposition and proved that *LGALS12* affected porcine intramuscular and subcutaneous adipogenesis via different signaling pathways [17]. All these data support the idea that *LGALS12* is a candidate gene for genetic improvement of fat-related traits in pigs.

Although it was demonstrated to be an adipocyte-specific gene, the transcriptional mechanism of *LGALS12* remains unclear. Here, we found that several transcription factors related to lipid metabolism play a pivotal role in *LGALS12* transcription. PPARγ, C/EBPβ and KLF15 could promote *LGALS12* transcription, while KLF4 had the opposite effect. The expression of C/EBPβ is an early event during the differentiation of adipocytes, and it can induce the expression of C/EBPα and PPARγ, which are the master transcription factors for adipogenesis. Since *LGALS12* was expressed after adipogenic induction, it is expected that the transcription of *LGALS12* is regulated by C/EBPβ and PPARγ. KLF15 and KLF4 belong to a family of zinc finger transcription factors, which play diverse roles during mammalian cell differentiation and development. KLF15 expression is markedly increased during the differentiation of 3 T3-L1 pre-adipocytes. Knockdown of *KLF15* reduced the expression of PPARγ and suspended adipogenesis in 3 T3-L1 cells. Ectopic expression of KLF15 in NIH 3 T3 or C2C12 cells induced PPARγ expression and promoted triglyceride accumulation when the cells were exposed to an adipogenic medium [18].. Adipose tissue-specific *KLF15* knockout (AK15KO) mice exhibited decreased adiposity and increased lipolysis. Moreover, AK15KO mice showed resistance to high-fat diet induced obesity and insulin resistance [19]. Mechanistic studies revealed that KLF15 regulates genes related to triglyceride synthesis and suppresses lipolysis. In this study, KLF15 led to the largest increase of *LGALS12* promoter activity, contributing new evidence for the role of KLF15 in adipocytes. However, KLF4 suppressed *LGALS12* promoter activity in this study. KLF4 was discovered in 1996 and was proved to be one of four factors required for the induction of pluripotent stem cells (iPSCs) in 2006 [20]. The function of KLF4 in adipocytes was not elucidated so far, but a study showed that KLF4 inhibits the differentiation of intramuscular preadipocytes by targeting C/EBPβ [21], which supports our finding that KLF4 can suppress adipogenesis.

We cloned a 4 kb fragment of the *LGALS12* promoter (L-4 kb) and proved its tissue-specific promoter activity in adipocytes using a dual-luciferase reporter assay. In order to test its function, L-4 kb was used to drive apolipoprotein R (*APOR*) gene expression in adipocytes. In a previous study, we found that overexpression of *APOR* could increase lipolysis in adipocytes [22], and adipose tissue-specific expression of pig *APOR* protected mice from diet-induced obesity. In this study, decreased triglyceride accumulation in cells and an increased level of non-ester fatty acids in the culture supernatant were observed, which showed that *APOR* expression driven by L-4 kb recapitulated its physiological role in 3 T3-L1 cells.

## Conclusions

In summary, the present study demonstrates that *LGALS12* is an adipose tissue-specific gene in pigs. Transcription factors that regulate the *LGALS12* promoter were identified and the 4 kb fragment of porcine *LGALS12* promoter exhibited adipocyte-specific promoter activity. Our finding provides new evidence for understanding porcine fat deposition and a promoter element for adipose tissue-specific genetic modification in pigs.

## Methods

### Animals

Three 4-month-old large white pig were provided by Zhejiang Huateng Agricultural Technology Co., Ltd. Pigs were dissected for sampling after euthanatizing in CO_2_ euthanasia box. The longissimus dorsi muscle, subcutaneous adipose tissues, cardiac apex, the right lobe of liver, spleen, right kidney, the upper lobe of the right lung and intestine tenue (jejunum) were collected in liquid nitrogen and stored at − 80 °C until RNA extraction. Jiaxing University Animal Care Committee approved and verified all the experimental procedures and followed ARRIVE guidelines to perform the experiments [23].

### Digital differential display (DDD) analysis

Digital Differential Display (DDD) is an algorithmic system for the identification of differentially expressed genes based on the relative abundance of expressed sequence tags (ESTs) from two or more contrasting cDNA libraries, which are deposited in the NCBI UniGene database (http://www.ncbi.nlm.nih.gov/UniGene/). DDD compares the number of assignments of ESTs from several different libraries, or pools of libraries, to a specific UniGene cluster. To account for the unequal number of ESTs in each library, DDD utilizes Fisher’s exact test to restrict the output to statistically significant differences (*P* ≤ 0.05) [9]. Gene expression levels of adipose tissue derived cDNA libraries (pool A) against the other organ-specific cDNA libraries (pool B including intestine, kidney, longissimus, lung, muscle, ovary, spleen, testis, thymus, uterus) were compared. Genes with statistically significant differential expression between adipose and non-adipose tissues were recorded. For these recorded genes, fold changes of expression levels were calculated by dividing the frequency of that gene in pool A (adipose tissue) to the frequency in pool B (non-adipose tissue). More than 10-fold changed genes were subjected to Gene Ontology (GO) and Kyoto Encyclopedia of Gene and Genome (KEGG) [24] pathway analysis using the online databases DAVID 6.8.

### Q-PCR

Total RNA was isolated from pig tissues using Trizol reagent (Invitrogen, USA). The HiFiScript gDNA Removal cDNA Synthesis Kit (CWBiotech, Beijing, China) was sued for cDNA synthesis. Q- PCR was conducted using the 2 × plus SYBR real-time PCR mixture (BioTek, Beijing, China) on a QuantStudio 3 system (Thermo fisher, USA). The primer sequences are listed in Table S1. Relative gene expression levels were calculated using the 2^*-ΔΔCt*^ method with GAPDH as the internal control.

### Promoter analysis

The BDGP [25] online tool was used to predict core promoter sequence, and the type of organism selects eukaryote and the threshold score was set by default to 0.8. The JASPAR [26] website was used to identify the transcription factor binding sites, and the taxonomic group was set to vertebrata.

### Vector construction

The pDsRed-Express-N1 vector was amplified by PCR using primers 5′- ATAGAGCTCCGCGG.

AACTCCATATATGGG-3′ and 5′-CTCGAGCTCAAGCTTCGAATTCTGC-3′, then digested by SacI and ligated to construct a new tool vector without CMV promoter. The *LGALS12* promoter (− 4061 to + 121) was cloned into the new vector digested by SacI and SalI, and the primers were 5′-ATAGAGCTCAGCATACATGGAATACATGCTG-3′ and 5′-ATAGTCGACGCCCAACTGAGCC.

CTGAGAC-3′, namely pDsRed-L-4 kb vector. Next, the pDsRed-L-4 kb vector and the *APOR* coding sequence was amplified using primers 5′-CAGTGGTACCCAAAGAAGAAAAGTGTCA.

G-3′ and 5′-CTAACCGGTTACAGCTCCAGGGCCAATTTTATCTCTCC-3′, were digested by KpnI and AgeI, then ligated to construct pDsRed-L-4 kb-*APOR* vector.

### Cell culture and transfection

Preadipocyte cell line 3 T3-L1 was purchased from ATCC, and cultured in DMEM containing 10% fetal bovine serum (FBS, Gibco, LOT2206993CP) to confluence (day 0), and then shifted to adipocyte differentiation medium, which was DMEM with 10% FBS, 0.5 mM dexamethasone, 20 nM insulin, and 0.5 mM isobutyl methylxanthine (IBMX) for 2 days (Day 3). From day 4 to day 8, cells were maintained in DMEM with 10% FBS and 20 nM insulin, and the medium was replaced every other day. Lipofectamine™ 2000 (11668027, Invitrogen, USA) was used for plasmid transfection at the indicated time points. Then, the medium was replaced with fresh medium in 24 hours.

### Luciferase reporter assays

HEK293T were seeded in 24-well plates and co-transfected with *LGALS12* promoter and transcription factor (PPARα、C/EBPα、CEBPβ、KLF4 and KLF15) vector by VigoFect (Vigorous Biotech, Beijing). The pGL3-Basic and pCR3.1 vector were used to insert promoter and transcription factor sequence, respectively. The pTK-Renila luciferase reporter (Promega) was included in all transfections for normalization. Luciferase activities were measured after transfection for 24 h using the dual-luciferase reporter assay system (Promega), and each combined set of vectors had three independent replicates. The measure was performed as described [27] with some modification. Tecan’s Spark multimode microplate reader was used to measure fluorescence intensity, and the comparison was conducted based on the ratio of firefly luciferase activity to Renilla luciferase activity.

### Oil red O staining

The matured 3 T3-L1 cells were washed with PBS and fixed with 4% paraformaldehyde for 30 min at room temperature, then washed three times with PBS. The fixed cells were then covered with a mixture of Oil Red O solution (0.6% Oil Red O dye in isopropanol) and water at a 6:4 ratio for 30 min, followed by washing four times with PBS, and images were captured under an optical microscope (Leica Microsystems, Germany).

### Statistical analysis

The data were obtained from at least three independent experiments, and presented as the means ± standard error (SE). GraphPad Prism 8.0 (GraphPad, San Diego, CA, USA) were used to conduct the statistical analysis, and the assessment of normality are Kolmogorov-Smirnov (K-S) test. Individual comparisons were assessed using Student’s t-test. *P*-values of less than 0.05 were considered to indicate significant differences, are displayed as * *p* < 0.05, ** *p* < 0.01, and *** *p* < 0.001.

## Supplementary Information

**Additional file 1: Table S1.** Primers for Q-PCR analysis.

**Additional file 2: Table S2.** Annotation information of 100 ESTs with expression levels 10-fold higher inadipose tissue than in non-adipose tissues.

## Data Availability

The NCBI UniGene database was inquired to analyze the relative abundance of ESTs, and other data sets supporting the results of this article are included within the manuscript and its additional files.

## Abbreviations

GM: genetically modified
CRISPR: Clustered Regularly Interspaced Short Palindromic Repeats
DDD: Digital differential display
Q-PCR: quantitative real time polymerase chain reaction
*LGALS12*: galectin 12
EST: Expressed Sequence Tag
GO: Gene Ontology
KEGG: Kyoto Encyclopedia of Genes and Genomes
IBMX: isobutylmethylxanthine
PPAR: peroxisome proliferator activated receptor
AMPK: adenosine 5′-monophosphate (AMP)-activated protein kinase
FABP4: fatty acid binding protein 4
ADIPOQ: adiponectin, C1Q and collagen domain containing
CIDEC: cell death inducing DFFA like effector c
LPL: lipoprotein lipase
LIPE: lipase E, hormone sensitive type
SDR16C5: short chain dehydrogenase/reductase family 16C member 5
SMAF1: Also known as ADIG, adipogenin
CEBPα: CCAAT enhancer binding protein alpha
C/EBPβ: CCAAT/enhancer binding protein (C/EBP), beta
PPARγ: peroxisome proliferator activated receptor gamma
KLF4: Kruppel like factor 4
KLF15: Kruppel like factor 15
*APOR*: apolipoprotein R
