# Supplementary material for: Cloning and functional verification of a porcine adipose tissue-specific promoter
Source: BMC Genomics. 2022 May 24;23:394. doi: 10.1186/s12864-022-08627-0 (PMC9128115; doi:10.1186/s12864-022-08627-0)
Supplement: Supplementary file 1 — Additional file 1: Table S1. Primers for Q-PCR analysis. [file 12864_2022_8627_MOESM1_ESM.pdf]

Table S1. Primers for Q-PCR analysis

Gene name	Forward primer (5' to 3')	Reverse primer (5' to 3')
GAPDH	F: ATCACCATCTTCCAGGAGCGA	R: AGCCTTCTCCATGGTCGTGAA
LGALS12	F1: CTCTTCTACCCCCAGCGATTCT	R1: ACTGATGTGCAGCTCTCGTAGC
	F2: CTTCTACCCCCAGCGATTCTT	R2: CTGATGTGCAGCTCTCGTAGC
ADIPOQ	F1: AGGTCTTACTGGTCCTAAGGGTGA	R1: TCTCCAGGTTCTCCTTTTCTGC
	F2: CGAGAAGGGTGAGAAAGGAGA	R2: GACCTTCAACCCCAGTCACTC
FABP4	F1: GTGCAGAAGTGGGATGGAAAG	R1: TTCTGGTAGCCGTGACACCTT
	F2: GCCAGGAATTTGATGAAGTCAC	R2: GGTTGTCTTTCCATCCCACTTC
CIDEC	F1: TCTACGGTGCCTACTCCCTTTC	R1: CAGGTAACAGGAGGTGCCAAG
	F2: CATCACAGCAGGGCACTAGGT	R2: TCAGGCAACCAATGAAGTCCT
SDR16C5	F1: ATGCCATCCTGAAAGAACAACT	R1: AATTAAAGGCACCCACGTACTC
	F2: TGCCATCCTGAAAGAACAACTG	R2: TGCCATCCTGAAAGAACAACTG
LPL	F1: CCGAGAGTGAAAACATCCCTTT	R1: TCACTGACCCATTTGAGTTTCA
	F2: GCCAAAAGAAGCAGCAAAATG	R2: GCCACGGTGCCATATAGAGAG
LIPE	F1: AACTTGGTGCCCACAGAAGAG	R1: GCTCAGGTCATGCAGTGTCAG
	F2: ACTCCTTCCTGGAGCTGAGTG	R2: CTTGAGAGAGTCCGTTCCCAGT
SMAF1	F1: ATCGTCTGGCTACGCTTCCTAC	R1: CTCTCCTCCTCTGGGCTATGAA
	F2: TGGCTACGCTTCCTACTCAGC	R2: TCTCCTCCTGGCAAAACTCAG
