# Supplementary material for: Cloning and functional verification of a porcine adipose tissue-specific promoter
Source: BMC Genomics. 2022 May 24;23:394. doi: 10.1186/s12864-022-08627-0 (PMC9128115; doi:10.1186/s12864-022-08627-0)
Supplement: Supplementary file 2 — Additional file 2: Table S2. Annotation information of 100 ESTs with expression levels 10-fold higher inadipose tissue than in non-adipose tissues. [file 12864_2022_8627_MOESM2_ESM.pdf]

Table S2. Annotation information of 100 ESTs with expression levels 10-fold higher in adipose tissue than in non-adipose tissues

UniGene ID	ESTs annotations	Gene symbol
Ssc.78659	Adiponectin, C1Q and collagen domain containing (ADIPOQ)	*ADIPOQ*
Ssc.1089	Fatty acid binding protein 4, adipocyte (FABP4)	*FABP4*
Ssc.6784	Lipase, hormone-sensitive (LIPE)	*LIPE*
Ssc.16335	Lipoprotein lipase (LPL)	*LPL*
Ssc.21815	Cell death-inducing DFFA-like effector c (CIDEC)	*CIDEC*
Ssc.49679	Lectin, galactoside-binding, soluble, 12 (LGALS12)	*LGALS12*
Ssc.87912	Small adipocyte factor 1 (SMAF1)	*SMAF1*
Ssc.48111	Short chain dehydrogenase/reductase family 16C, member 5 (SDR16C5)	*SDR16C5*
Ssc.12241	Annexin A2 (ANXA2)	*ANXA2*
Ssc.16159	Stearoyl-CoA desaturase (delta-9-desaturase) (SCD)	*SCD*
Ssc.10451	Serpin peptidase inhibitor, clade F (alpha-2 antiplasmin, pigment epithelium derived factor), member 1 (SERPINF1)	*SERPINF1*
Ssc.79705	Calpain, small subunit 1 (CAPNS1)	*CAPNS1*
Ssc.1342	Apolipoprotein E (APOE)	*APOE*
Ssc.13774	Ribosomal protein S12 (RPS12)	*RPS12*

Ssc.1051	Retinol saturase (all-trans-retinol 13,14-reductase) (RETSAT)	*RETSAT*
Ssc.638	Ubiquitin A-52 residue ribosomal protein fusion product 1 (UBA52)	*UBA52*
Ssc.51793	Ribosomal protein, large, P1 (RPLP1)	*RPLP1*
Ssc.5943	Biglycan (BGN)	*BGN*
Ssc.55440	Peptidylprolyl isomerase A (cyclophilin A) (PPIA)	*PPIA*
Ssc.18175	Fatty acid synthase (FASN)	*FASN*
Ssc.32545	Small EDRK-rich factor 2 (SERF2)	*SERF2*
Ssc.1320	Lectin, galactoside-binding, soluble, 1 (LGALS1)	*LGALS1*
Ssc.20399	Cell death-inducing DFFA-like effector a (CIDEA)	*CIDEA*
Ssc.17347	Pyruvate carboxylase (PC)	*PC*
Ssc.15379	Diacylglycerol O-acyltransferase 2 (DGAT2)	*DGAT2*
Ssc.19566	Secreted frizzled-related protein 4 (SFRP4)	*SFRP4*
Ssc.70048	1-acylglycerol-3-phosphate O-acyltransferase 1 (lysophosphatidic acid acyltransferase, alpha) (AGPAT1)	*AGPAT1*
Ssc.2647	Adiponectin receptor 2 (ADIPOR2)	*ADIPOR2*
Ssc.55858	Diazepam binding inhibitor (GABA receptor modulator, acyl-CoA binding protein) (DBI)	*DBI*
Ssc.2257	Non-metastatic cells 2, protein (NM23B) expressed in (NME2)	*NME2*

Ssc.53748	Ribosomal protein L23 (RPL23)	*RPL23*
Ssc.16327	Collagen, type V, alpha 3 (COL5A3)	*COL5A3*
Ssc.5936	Translocator protein (18kDa) (TSPO)	*TSPO*
Ssc.16650	Branched chain keto acid dehydrogenase E1, alpha polypeptide (BCKDHA)	*BCKDHA*
Ssc.947	Ribosomal protein S28 (RPS28)	*RPS28*
Ssc.94127	Fatty acid binding protein 5 (psoriasis-associated) (FABP5)	*FABP5*
Ssc.94399	MHC class I antigen 5 (SLA-5)	*SLA-5*
Ssc.50776	Collagen, type VI, alpha 1 (COL6A1)	*COL6A1*
Ssc.1942	Lipin 1 (LPIN1)	*LPIN1*
Ssc.51886	Microsomal glutathione S-transferase 1 (MGST1)	*MGST1*
Ssc.6947	ATP synthase, H+ transporting, mitochondrial F1 complex, epsilon subunit (ATP5E)	*ATP5E*
Ssc.15742	Cytochrome P450 2A19 (CYP2A19)	*CYP2A19*
Ssc.59677	Perilipin 1 (PLIN1)	*PLIN1*
Ssc.16018	Acetyl-CoA carboxylase alpha (ACACA)	*ACACA*
Ssc.1187	Fructose-bisphosphate aldolase C-like (LOC100512013)	*ALDOC*
Ssc.6473	Transcribed locus, strongly similar to NP_062110.1 procollagen C-endopeptidase enhancer 1 [Rattus norvegicus]	*RACK1*

Ssc.46811	Collagen alpha-1(I) chain-like (LOC100516693)	*COL1A1*
Ssc.56487	Transcribed locus, moderately similar to NP_001090896.1 MHC class I antigen 3 [Sus scrofa]	*SLA-1*
Ssc.19490	60S ribosomal protein L23a-like (LOC100514795)	*RPL23A*
Ssc.53718	60S acidic ribosomal protein P2-like (LOC100513643)	*RPLP2*
Ssc.1050	Transaldolase-like (LOC100514210)	*TALDO1*
Ssc.8706	Regulator of G-protein signaling 3-like (LOC100517801)	*RGS3*
Ssc.33496	Gelsolin (LOC396874)	*GSN*
Ssc.78646	Transcribed locus, weakly similar to XP_593741.4 PREDICTED: hypothetical protein [Bos taurus]	*CYP11A1*
Ssc.53720	ELMO domain-containing protein 3-like (LOC100519304)	*ELMOD3*
Ssc.18549	Adiponectin, C1Q and collagen domain containing (ADIPOQ)	*ADIPOQ*
Ssc.3648	Transmembrane protein 140-like (LOC100523705)	*TMEM140*
Ssc.55000	Gasdermin-D-like (LOC100515607)	*GSDMD*
Ssc.55239	Transcribed locus, strongly similar to NP_999411.1 liver carboxylesterase [Sus scrofa]	*CES1*
Ssc.55987	Transcribed locus, strongly similar to XP_904556.1 PREDICTED: similar to hCG2016250, isoform 3 [Mus musculus]	*EG625055*
Ssc.17921	Transcribed locus, strongly similar to XP_001927405.1 PREDICTED: immunoglobulin-binding protein 1 isoform 1 [Sus scrofa]	*IGBP1*
Ssc.53744	Hypothetical protein LOC100524865 (LOC100524865)	*ZC3H10*

Ssc.19211	Cytochrome P450 4B1-like (LOC100511024)	*CYP4B1*
Ssc.51902	Dermatopontin-like (LOC100516366)	*DPT*
Ssc.24163	40S ribosomal protein S25-like (LOC100516385)	*RPS25*
Ssc.49339	Progestin and ADIPOQ receptor family member 6-like (LOC100157531)	*PAQR6*
Ssc.53724	Transcribed locus, strongly similar to NP_956321.1 60S ribosomal protein L10 [Danio rerio]	*RPL10*
Ssc.53705	60S ribosomal protein L32-like (LOC100526011)	
Ssc.760	Liver carboxylesterase-like (LOC100517530)	*CES1*
Ssc.9637	Transcribed locus	
Ssc.26980	Basal cell adhesion molecule-like (LOC100512053)	*CYP11A1*
Ssc.74973	Protein S100-A1-like (LOC100524861)	*SNTN*
Ssc.5576	Transcribed locus, strongly similar to NP_001020068.1 EH domain-containing protein 2 [Rattus norvegicus]	*EHD2*
Ssc.55121	ORM1-like protein 3-like (LOC100511588)	*ORMD13-L*
Ssc.49801	Transcribed locus, strongly similar to NP_001098513.1 dipeptidyl peptidase 7 [Bos taurus]	*DPP7*
Ssc.8567	Transcribed locus, strongly similar to NP_000987.2 60S ribosomal protein L35a [Homo sapiens]	*RPL35A*
Ssc.37612	Transcribed locus, strongly similar to NP_001033727.1 perilipin-1 [Sus scrofa]	*PLIN1*
Ssc.26906	3-ketoacyl-CoA thiolase, peroxisomal-like (LOC100517370)	*ACAA1*

Ssc.8046	Peptidyl-prolyl cis-trans isomerase A-like (LOC100153727)	
Ssc.50103	Transcribed locus, strongly similar to XP_003128776.1 PREDICTED: tryptophanyl-tRNA synthetase, cytoplasmic-like [Sus scrofa]	
Ssc.43763	Transcribed locus, strongly similar to NP_277053.2 sodium-coupled neutral amino acid transporter 5 [Homo sapiens]	*SLC38A5*
Ssc.56841	Transcribed locus, strongly similar to NP_001839.2 collagen alpha-1(VI) chain precursor [Homo sapiens]	*COL6A1*
Ssc.72012	Transcribed locus, strongly similar to NP_001003447.1 60S ribosomal protein L15 [Danio rerio]	*RPL15*
Ssc.11742	Transcribed locus, strongly similar to NP_032188.3 phospholipid hydroperoxide glutathione peroxidase, nuclear isoform 2 precursor [Mus musculus]	*GPX4*
Ssc.20228	U4/U6 small nuclear ribonucleoprotein Prp31-like (LOC100520791)	*PRPF31*
Ssc.23336	Endothelial cell-selective adhesion molecule-like (LOC100522817)	*ESAM*
Ssc.1154	60S ribosomal protein L36-like (LOC100512647)	*DUT*
Ssc.15701	Transcribed locus, strongly similar to XP_213746.1 PREDICTED: similar to tetratricopeptide repeat domain 11 [Rattus norvegicus]	*FIS1*
Ssc.41844	Fatty acid-binding protein, heart-like (LOC100522323)	
Ssc.56803	Transcribed locus, moderately similar to NP_001075222.1 elastin isoform c precursor [Homo sapiens]	*ELN*
Ssc.56462	Transcribed locus, strongly similar to NP_033106.1 60S ribosomal protein L26 [Mus musculus]	*RPL26*
Ssc.56953	Transcribed locus, strongly similar to NP_990284.1 ubiquitin-40S ribosomal protein S27a [Gallus gallus]	*RPS27A*
Ssc.32472	Thyroid hormone-inducible hepatic protein-like (LOC100511213)	*THRSP*
Ssc.44970	6.8 kDa mitochondrial proteolipid-like (LOC100523804)	*ATP5MJ*

Ssc.53877	Transcrib+A101+B97	*RPL37*
Ssc.93967	Cytochrome P450 4B1-like (LOC100523909)	*CYP4B1*
Ssc.20344	WW domain-binding protein 2-like (LOC100521149)	*WBP2*
Ssc.39642	Transcribed locus, moderately similar to NP_001096815.1 butyrophilin-like 9 [Bos taurus]	*BTNL9*
Ssc.49841	Transcribed locus	
